# Fatty Acid Composition of Phospholipids and in the Central and External Positions of Triacylglycerol in Muscle and Subcutaneous Fat of Beef Steers Fed Diets Supplemented with Oil Containing n6 and n3 Fatty Acids While Undergoing One of Three 48 h Feed Withdrawal Treatments

**DOI:** 10.1155/2012/543784

**Published:** 2012-07-29

**Authors:** C. Margetak, G. Travis, T. Entz, P. S. Mir, S. Wei, M. V. Dodson

**Affiliations:** ^1^Agriculture and Agri-Food Canada, 5403 1st Avenve South P.O. Box 3000, Lethbridge, AB, Canada T1J 4B1; ^2^Department of Animal Sciences, Washington State University, Pullman, WA 99163-646351, USA

## Abstract

This study was designed to determine the effects of dietary oil and feed withdrawal treatments on fatty acid composition of phospholipids of triacylglycerol in pars costalis diaphragmatis muscle and subcutaneous fat from the brisket. A 2 × 3 factorial experiment was conducted with crossbred steers with an initial body weight of 280.5 ± 5.8 kg. Steers were fed either a control or an oil containing diet where 5% of the control diet was replaced with an equal mixture sunflower and flax oil while undergoing one of three feed withdrawal treatments: no withdrawal, a single 48 h withdrawal before initiation of fattening at one year of age, or 48 h withdrawal at 8 wk intervals from weaning to initiation of fattening. At time of processing samples of muscle and fat were obtained and analyzed to determine fatty acid composition. Disproportionate distribution of the fatty acids was observed by diet, feed withdrawal regimen and whether the sample was from muscle or fat. Differences are discussed in detail, and our data suggests a special function for the fatty acids that accumulate in specific positions of the triacylglycerol due to treatment.

## 1. Introduction

The discovery of the anticarcinogenic, antiadipogenic, and anti-inflammatory properties of fatty acids led to studies to enhance the occurrence of these functional fatty acids such as conjugated linoleic acid (CLA) in human foods. Enhancing the naturally occurring CLA in ruminant products to provide naturally formed CLA as opposed to synthesized CLA to produce a value added agricultural product was among the objectives. However, concerns have been raised [1] that the efficacy of synthetic CLA may be marginal with regards to decreasing the size of adipose tissue in humans relative to that in other species (swine; [2]) and that the extent of the increase in natural products may be less adequate to meet the requirements. Yet, the discovery of the anticarcinogenic properties CLA was from beef extracts [3], which were perhaps not particularly in high concentration. When Gaullier et al. [4] compared the effects of CLA free fatty acid and CLA- triacylglycerol (TAG), they found that while both forms of CLA were effective over the 12 m period of the study. Although a difference between the free fatty acid and the TAG was not significant, the CLA-TAG consistently resulted in greater weight loss and decreases in body mass index and body fat mass. Similarly, either a decrease in inguinal fat content [5] or a decrease in fat cell number in the inguinal fat was noted [6], in rats fed beef from cattle fed dietary oil to increase the CLA content of the beef fat, even though the amount of CLA provided from the beef was lower than the amount of synthetic CLA in the test diet. These observations appear to suggest that the consistent positive effect of the CLA in triacylglycerol (synthetic compound or as beef fat) may be related to the CLA at the sn2 position. The fatty acid in the sn2 position is not hydrolysed by either pancreatic lipase or lipoprotein lipase in the digestive tract or extrahepatic tissues, respectively [7], but is carried to the liver for further metabolism [8]. Chardigny et al. [9] studied the location of labelled CLA in TAG at either the sn1/3 or sn2 position, and found that the label was recovered in oxidation products when CLA was in the sn1/3 position but was found in the carcass when the labelled CLA was in the sn2 position. These authors found that dairy CLA was uniformly distributed among the three positions of milk fat, as did Paterson et al. [10], who found that bioformed CLA from sheep fed safflower oil aggregated to greater proportions at the sn1/3 position in muscle fat. However, Mir et al. [11] found that muscle fat from beef steers fed sunflower oil had greater proportions of CLA at the sn2 position, but the amounts were greater when the dietary oil incorporation level was 3% of diet rather than 6%. In order to extend the understanding of the effects of diet and feed withdrawal (FW) on fatty acid distribution to phospholipid (PL) or TAG and in TAG to the sn2 or sn1/3 positions, the fatty acid composition of PL, TAG, and sn2 in fat from muscle (*Pars costalis diaphragmatis*; PCD) and subcutaneous (SQ) fat needed determination. The present study was conducted with the objective of determining the fatty acid composition of phospholipids (PL), TAG and sn2 position and to calculate the proportion of the fatty acids at the sn1/3 position. The fat was obtained from steers in an experiment [12] where they were fed either a control (CON) or an oil (OIL) diet where 5% of the diet was replaced with equal amounts of sunflower and flax oils. These diets were fed to steers in three FW treatments, no FW, single FW (FW × 1), which occurred at yearling age for 48 h and a multiple FW (FW × 4), where the steers were denied feed for 48 h every eight weeks between weaning and one year of age. 

## 2. Materials and Methods

### 2.1. Animals and Diets

 A total of 72, spring born, European crossbred (with Hereford, Angus, and Charolais genetics), steer calves were obtained upon weaning (280.5 ± 5.8 kg) and housed in the Individual Feeding Barn of the Lethbridge Research Centre following the guidelines of the Canadian Council on Animal Care [13]. The study was started after receiving approval of the Institutional Animal Care Committee (Approval no. 0727). The vaccination protocol, treatment assignment, and diets fed to the steers have been provided in He et al. [12]. The six treatments applied to the steers in a 2 × 3 factorial arrangement (Figure 1, [14]; in press), where each of two diets was provided to the steers undergoing one of three FW treatments, where each FW lasted for 48 h, but water was always available.

The steers were fed either the CON or OIL diet. In the OIL diet 5% of the diet was replaced with an equal mixture of flax and sunflower oil. The oil replaced the steam rolled barley in the diet [12]. The FW treatments were no FW, single FW (FW × 1 [15]) for 48 h at yearling age, just before initiation of the fattening phase, or 48 h FW every 8 wk, which occurred four times (FW × 4), between start of the experiment and until they were approximately a year in age, and before the transition to the fattening phase was initiated. Each FW was started on the weigh day after recording the BW and the feed bunks were cleaned for all the steers. Only those predetermined to undergo FW did not receive the feed after the BW was recorded, while steers in other treatments were provided feed. After the 48 h of FW, the steers in all treatments were provided with the respective diets. 

The animals were fed once daily with total mixed rations. Animals were weighed every 4 wk through the growing phase and every 3 wk during the fattening phase. At the end of the trial when steers were judged visually by the commercial abattoir purchaser as carrying adequate fat to yield 57% lean meat, the animals were weighed on full feed on two consecutive days and shipped on full feed and were processed at a commercial abattoir [12]. At processing, samples of PCD and SQ from each animal were collected and placed on ice and transported to the laboratory. Fat from the PCD and SQ was extracted as described in He et al. [12]. The fat was separated into triacylglycerol (TAG) and phospholipids (PL), and the TAG from each sample was digested so that the monoacylglycerol (sn2MAG) could be collected and the fatty acid composition of each fraction could be determined. The relative fatty acid composition of sn2 and that at the sn1/3 positions of the TAG was calculated from the fatty acid composition of the TAG and the sn2.

### 2.2. Separation of Lipid Classes

Total lipid was fractioned into TAG and PL using column chromatography to separate the fractions [16]. Standard TAG and PL were separated by column chromatography, and the separated fractions were then resolved by thin layer chromatography to ascertain the fractions and that separation was appropriate. The affirmation of separation was performed by using TLC (Analtech Uniplate Silica Gel 250 *μ*m plates, 20 × 20 cm; 75 Blue Hen Drive, Newark, DE, 19713), with hexane/diisopropyl ether (75 : 25; v/v) as the solvent. Separations were compared with 20 *μ*L of 10 mg/mL trioleoylglycerol (Sigma-Aldrich Canada, Oakville, ON, Canada) plated as a reference standard. Briefly, the fractions were separated using silica gel columns, which were constructed using 10 mL micropipette tips. Approximately 1.3 g of conditioned silica gel (Alltech 63–200 *μ*m; 2051 Waukegan Road Deerfield, IL, 60015) dried overnight at 160°C then mixed with ultra high purity (UHP) water in a 95 : 5 ratio and sandwiched between conditioned cotton (cotton was soaked in CHCl_3_ : CH_3_OH :  C_6_H_14_ 1 : 1 : 1 overnight, changing solvent every 8 h, then air dried). A solution of fat from each sample in toluene to a concentration of 100 mg/mL was prepared and 1 mL was loaded on to the column. Immediately following the loading of the sample onto the column, 1 mL of solvent (hexane/diisopropyl ether 85 : 15 v : v for PCD samples and 80 : 10 v : v for SQ) was added to the column and held for exactly two minutes. For samples from the PCD, a 2 mL initial solvent rinse following the loading phase was required to remove unwanted residue. Following the solvent wash (to discard the residue) for the PCD, all fat samples were eluted with two, 3 mL portions of the respective solvents. After elution, the tip was washed with 400 *μ*L of toluene. All portions of the elution were collected into a preweighed glass vial. Solvent in the vial was later dried under a stream of N and the weight of TAG was recorded. 

Further, the PL was retrieved from the column by eluting the column with two 3 mL rinses of di-isopropyl ether as the solvent for the PCD samples and methanol for the SQ samples. The eluted solvent with the PL from the sample was collected into a preweighed glass vial and solvent was removed under a stream of N. All TAG and PL samples were stored in CHCL_3_ at −20°C. 

#### 2.2.1. Digestion of TAG with Pancreatic Lipase

Pancreatic lipase (Lipase from porcine pancreas, Type II; Sigma-Aldrich Canada, Oakville, ON) was used to generate sn2-MAG from TAG [10]. Two milligrams of dried TAG wERE suspended in 500 *μ*L of pancreatic lipase buffer [1 M Tris-HCl, pH 8, containing 10% gum Arabic (wt/v) and 0.23 M CaCl_2_ (wt/v)] by sonication. Exactly 500 *μ*L of pancreatic lipase buffer containing 8 mg pancreatic lipase/mL was added to the TAG suspension; the mixture was vortexed for 30 s and incubated at 37°C for 1 hr in a shaking water bath. The reaction was stopped with 500 *μ*L of 0.1 N acetic acid, and the lipid was extracted three times with 2 mL diethyl ether. Each extract was passed through a small column of anhydrous Na_2_SO_4_, combined, and then evaporated to dryness under N. The extracted lipid was redissolved in 200 *μ*L CHCl_3_ and applied to boric acid TLC plates (Analtech Silica Gel G 5% (wt/v) boric acid, 250 *μ*m plates, 20 × 20 cm; 75 Blue Hen Drive, Newark, DE, 19713). A reference standard of 20 *μ*L of 20 mg/mL of 2-Oleoylglycerol (Sigma-Aldrich Canada, Oakville, ON, Canada) in CHCl_3_ solution was applied to each plate. In order to have adequate sample, two spots were applied for each sample. Lipids were separated by one ascension of CHCl_3_/CH_3_COCH_3_ (88/12, v/v). The sn2-MAG standard was visualized using iodine vapor, so that the sn2 MAG from the samples could be detected for elution and collection.

#### 2.2.2. Elution of Monoacylglycerol from Silica Gel

The silica gel containing the fraction of interest (sn-2 MAG) was removed from the TLC plate using a razor blade scraper and then transferred into methanol-washed test tubes (a tube for each sample). Lipids were eluted from the silica by extracting twice with 5 mL and once with 2 mL of CHCl_3_. The slurry was shaken vigorously and centrifuged at 400 ×g for 3 min. The eluted solvent with the sn2-MAG was passed through a column of anhydrous Na_2_SO_4_. The sn2-MAG separated by thin layer chromatography from the two applications for each sample were combined and dried under N and then stored in 1 mL of toluene under N at −20°C.

### 2.3. Fatty Acid Analysis 

#### 2.3.1. Methylation of Samples

Samples of sn2-MAG, PL, and TAG were thawed and allowed to equilibrate to room temperature, then 10 *μ*L of 5.96 mg/mL C 19 : 0 was added as an internal standard and the samples were methylated [17]. Briefly, 1 mL of sodium methoxide (0.5 M) was added to the sn2-MAG samples, while 2.5 mL of sodium methoxide were added to samples of TAG, vortexed for 30 seconds, and then placed in a water bath at 50°C for 10 min. The partially methylated samples were removed and cooled to room temperature. To the cooled sample 0.5 mL or 1 mL Boron trifluoride (14% in CH_3_OH) was added to sn2-MAG and TAG samples, respectively, followed by vortexing for 30 seconds and then returned to the water bath at 50°C for an additional 10 min. After which the samples were removed, and cooled to room temperature and 2.5 and 5 mL UHP water was added to the sn2-MAG and TAG samples. The samples were vortexed, then 2.5 and 5 mL Hexane were added to sn2-MAG and TAG samples, respectively, and vortexed for 15 s. The hexane layer was allowed to separate and was transferred into autosampler vials, capped, and stored at −20°C.

#### 2.3.2. Gas Chromatography

 The methylated sn2-MAG, PL, and TAG were quantified by a gas-liquid chromatograph (GC System 6890, Hewlett-Packard, Mississauga, ON, Canada) equipped with a flame ionization detector and an SP-2560 fused-silica capillary column (100 m with 0.2 mm film thickness; Supelco Inc., Oakville, ON, Canada). Samples were loaded onto the column via 1 *μ*L splitless injections [18]. The parameters for separation are as provided by He et al. [12]. The composition of sn2-MAG and TAG for each fatty acid was calculated from the formula of Paterson et al. [10] where Sn1/3 wt% = (TAG wt%  × 3−sn2-MAG wt%)/2.

### 2.4. Statistical Analysis

 Data from the experiment were analysed by using PROC MIXED [19] as a completely randomised design. The treatment arrangement was as a 2 × 3 factorial experiment with each animal as the experimental unit and the treatment factors were the two diets and the three FW treatments. All values are provided as mean ± sem and differences among treatments were declared as significant at *P* < 0.05 and 0.05 < *P* < 0.1 was considered a trend. Differences between proportion of a fatty acid at sn2 and sn1/3 or PL and TAG were determined as difference between two means with unequal variances [20].

## 3. Results and Discussion

 The fatty acid composition of the fat from the PCD and SQ has been reported previously [12]. It was noted that although no trans C18:1, C18:2, CLA or elongated n3 fatty acids occurred in the diet, substantial amounts were found in muscle and SQ, thus their positional occurrence was of interest.

### 3.1. Pars Costalis Diaphragmatic

#### 3.1.1. Saturated Fatty Acids

 The proportional composition of the saturated fatty acids, C14:0, C15:0, C16:0, C18:0, and C20:0, in PL, TAG, and in sn2 and sn1/3 of fat from PCD, is presented in Table 1. Diet or treatment did not affect the proportions of C14:0, but greater (*P* < 0.05) proportions of this fatty acid were found in TAG than PL and at the sn1/3 than the sn2 position. Although C15:0 was present in only small amounts in the fat of the PCD, greater (*P* = 0.0545) proportions were observed in the PL of CON fed steers than those fed the OIL diet. Further, more (*P* < 0.05) C15:0 occurred in PL than TAG and at sn2 than the sn1/3 position. Dietary OIL suppresses de nova synthesis [21, 22] of C16:0, thus lesser (*P* = 0.001 to 0.0001) proportions of this fatty acid were observed in PL, TAG, and at the sn1/3 position, but diet did not affect the proportion of this fatty acid at the sn2 position and the fatty acid was distributed evenly between PL and TAG and sn2 and sn1/3. Contrary to C16:0, C18:0 in the fat from the PCD was greater (*P* = 0.004 to 0.0001) in the PL, TAG, and sn1/3 position of steers fed the OIL diet. Although a treatment effect was not observed for sn2, greater (*P* < 0.05) proportions of C18:0 were noted at the sn2 position than at the sn1/3 in steers fed the CON diet but not in those fed the OIL diet, which agrees with observations for beef fat [23]. The occurrence of C20:0 in PL of muscle of OIL fed steers was greater (*P* = 0.0307) than that in CON fed steers. The proportions of C20:0 at the sn2 position were greater (*P* < 0.05) than at sn1/3 and FW increased (*P* = 0.0391) this fatty acid at the sn2 location. The cause for this effect is not known, although this fatty acid occurs mainly in animals and the significance of its position in the TAG needs further study.

#### 3.1.2. Unsaturated Fatty Acids

Table 2 shows the composition of the unsaturated fatty acids in fat from the PCD, of steers and the proportional distribution between PL and TAG and at the sn2 and sn1/3 positions is provided. The OIL diet fed to steers decreased (*P* = 0.0133 to 0.0042) C16:1c9 in PL, TAG, and at the sn1/3 position without affecting the sn2 position. However, the proportion of C16:1c9 at the sn2 position was lesser (*P* < 0.05) than that noted for the sn1/3 position and is similar to that reported previously [23]. C16:1c9 increase was noted in PL of fat from the PCD in steers that underwent FW (*P* = 0.0095).

Dietary OIL elevated (*P* = 0.0001) C18:1t9 in the PL, TAG, and at the sn1/3 position, with greater (*P* < 0.05) proportions in PL than in TAG in OIL fed steers and at sn2 versus sn1/3 in CON fed steers. Although C18:1t11 was found in relatively greater (*P* = 0.0001) abundance in PCD of OIL fed steers, a preferred location with regard to PL or TAG and sn2 or sn1/3 was not observed in CON fed steers, but in OIL fed steers a greater proportion was noted in the TAG and sn1/3 positions relative to PL and sn2, respectively. Interactions (*P* = 0.0557 and 0.0493) between diet and FW treatments were observed for C18:1c9 for PL and fatty acid at the sn1/3 in the fat from the PCD because of decreases in this fatty acid in steers fed OIL in the FW × 4 treatment relative to that in steers fed the CON diet in the FW × 4 treatment. Substantially greater (*P* < 0.05) proportions of the fatty acid occurred at sn1/3 than at sn2 as was also noted in MUFA1 beef fat in the study by Smith et al. [23].

 The C18:2t9c11 was not found at the sn2 position, but feeding the OIL diet resulted in increasing (*P* = 0.0001) this fatty acid in PL, TAG, and sn1/3, with similar proportions being distributed to PL and TAG. Feeding the OIL diet to steers increased (*P* = 0.0406 to 0.0001) C18:2n6 and C18:3n3 in PL, TAG, and sn1/3 without affecting sn2, but relatively greater (*P* < 0.05) proportions of C18:2n6 were in PL than in TAG and at the sn2 position than at the sn1/3 in concurrence with previous studies [23]. With regard to the C18:3n3, its accumulation at the sn2 was greater (*P* < 0.05) than at the sn1/3, but differences in distribution between PL and TAG were not observed.

 The CLAc9t11 was greater (*P* = 0.0034 and 0.01 77) in PL and at the sn1/3 position in PCD of OIL fed steers, while an interaction (*P* = 0.0022) was observed for CLAc9t11 in TAG where FW × 1 in OIL fed steers led to increases relative to that in CON fed steers in the FW × 1 treatment. A preference for PL relative to TAG was not noted for CLAc9t11, which is similar to published data for lambs fed safflower oil [10] but unlike their observation, a greater (*P* < 0.05) proportion of CLAc9t11 was found at the sn2 position, which concurs with observations in steers fed sunflower oil at the 3% of diet level [11]. Similarly, CLAt10c12 was found to favour (*P* < 0.05) the sn2 position relative to the sn1/3 position.

### 3.2. Subcutaneous Fat 

#### 3.2.1. Saturated Fatty Acids

 In the SQ, tissue diet or FW treatments did not influence distribution of either C14:0 or C15:0 (Table 3) as was observed for the PCD; C15:0 was found at higher (*P* < 0.05) proportions at the sn2 position relative to the sn1/3 position. Decreases (*P* = 0.0022 and 0.0001) in C16:0 were observed in PL and TAG of the SQ fat of steers fed the OIL diet, but differences in distribution between PL and TAG or sn2 and sn1/3 were not present. Feeding the OIL diet to the steers increased (*P* = 0.0001) the C18:0 content in PL and TAG, but the relative proportions of C18:0 in sn2 and sn1/3 were decreased (*P* = 0.0275) and increased (*P* = 0.0521), respectively, when the OIL diet was fed to steers in the FW × 4 relative to those fed the CON diet in the same FW treatment. Further C18:0 tended to be higher (*P* < 0.05) at the sn2 position relative to the sn1/3, as observed previously [23], and to TAG relative to PL. Diet or FW effects were not observed for C20:0, but greater (*P* < 0.05) proportions were noted in the sn2 position of the SQ fat than at the sn1/3 position.

#### 3.2.2. Unsaturated Fatty Acids

 In the SQ fat, feeding the OIL diet to the steers led to decreases (*P* = 0.0712 to 0.039) of C16:1c9 in PL, TAG, and sn1/3, with greater (*P* < 0.05) proportions in the PL and sn1/3 position relative to TAG and sn2, respectively (Table 4). In steers fed the OIL diet, the SQ fat had elevated (*P* = 0.0001) levels of C18:1t9 and C18:1t11 in PL, TAG, and at sn1/3, but in OIL fed steers in the FW × 4 treatment greater (*P* = 0.042 and 0.003, resp.) proportion of the two fatty acids was found at the sn2 position than in CON fed steers. The C18:1c9 was increased in SQ of steers in the FW × 4 relative to those in the no FW treatment. Generally, as previously reported [11], C18:1c9 appeared in TAG and at the sn1/3 position to a greater (*P* < 0.05) extent relative to PL or the sn2 position. 

As observed in the fat from the PCD of the steers, C18:2t9c11 was not found in the sn2 position, but steer SQ fat was responsive to dietary oil and elevated (*P* = 0.0001) levels of the fatty acid were present in the PL, TAG, and sn1/3, with greater (*P* < 0.05) proportions being found in the PL, which was different from the observation in the PCD fat. The C18:2n6 was increased (*P* = 0.0262) in steers fed the OIL diet in the TAG and not in the PL, which is different from what was observed in the fat from the PCD. Greater (*P* < 0.05) proportions of the fatty acid were found in the sn2 than at the sn1/3 position. Furthermore, C18:2n6 was elevated (*P* = 0.0079) at the sn2 position in SQ fat of steers in fed the OIL diet, when they were in the FW × 4 treatment relative to that of steers fed the CON diet in the same FW treatment. The C18:3n3 fatty acid was increased (*P* = 0.0002 and 0.0001) due to dietary oil in the PL, TAG, but interactions were observed for the proportions of this fatty acid at the sn2 (*P* = 0.0021) and sn1/3 (*P* = 0.0153) positions due to different effects in steers in the FW × 4 treatment. Generally, greater (*P* < 0.05) proportions of the fatty acid were found in PL and at the sn2 position. The differences in composition of the PL with regard to composition of C18:2 n6 acids and diet are in concurrence with those of Dannenberger et al., [24] for phosphatidylcholine from concentrate or pastured cattle, while in contrast to their results for C18:3 n3 fatty.

Interactions (*P* = 0.0022 to 0.0475) were noted for CLAc9t11 for PL and TAG and for distribution at sn2 and sn1/3, which was largely due to differential effects in steers in the FW × 1 treatment. The CLAt10c12 was elevated in PL in SQ fat of OIL fed steers and was present in greater (*P* < 0.05) proportions in PL relative to TAG. Although greater (*P* < 0.05) proportions of this fatty acid occurred in the sn2 than at the sn1/3 position, interactions (*P* = 0.0174 and 0.0155) were observed for the distribution at the sn2 and sn1/3 position due to the effect of FW × 4 and FW × 1, respectively. These observations are in contrast to reports of Chardigny et al., [9] for milk where CLA was distributed largely to sn1/3.

### 3.3. Elongated n3 Fatty Acids in Muscle and Subcutaneous Fat

Table 5 summarises the distribution of EPA and DHA in the PCD and SQ fat of steers fed the CON or OIL diet in the FW treatments. However, neither diet nor FW affected either of these fatty acids except for DHA in SQ at the sn2 position where an interaction (*P* = 0.0225) was observed due to the differential effects of FW × 4 in steers fed the two diets. In both tissues, both EPA and DHA were found to greater (*P* < 0.05) extents in the PL and at the sn2 position relative to that in the TAG and at the sn1/3 position. In concurrence with the previous results, these elongated fatty acids diet only marginally affected PL composition [24] despite the strong effect on composition of the sn2 position. 

In general, it can be agreed that most unsaturated fatty acids favour the sn2 position [25], but C16:1 and C18:1c9 were found to occur in the sn1/3 and the fatty acid distribution results have been summarized in Table 6. Unlike the reports of Chardigny et al. [9] and Paterson et al. [10], CLA fatty acids occurred in the sn2 and not in the sn1/3 position in beef fat from PCD or SQ, which is in concurrence with reports of Mir et al. [11]. This difference in location in butter fat relative to beef fat may be contributory to the absence of effect on body composition in men provided butter with elevated levels of CLA [26]. The relative greater appearance of the two CLA fatty acids at the sn2 position in beef may signal a difference in efficacy, at lower concentrations as has been observed in rat studies with regard to effects on inguinal fat [5]. Further, the consistent, greater, although nonsignificant, effect of CLA triacylglycerol on body composition parameters relative to the free fatty acid [4] may be due to the effect of the CLA moiety at the sn2 position and its resistance to hydrolysis in the intestine [7] and its retention in the body [9]. 

## 4. Conclusion

Data from the study clearly indicate that provision of oil in the diet affected PL, TAG, and sn1/3 fatty acid composition to a greater extent than it did to that of the sn2 position. The independence of the fatty acid composition of the sn2 position suggests that the position attracts only certain number of each type of fatty acid. It was only in the case of the two CLA that an interaction between diet and FW was observed and FW × 4 was found to elevate these fatty acids at the sn2 location in steers fed the OIL diet. Usually increases of a fatty acid at the sn1/3 position led to increases of the fatty acid in the TAG. Although diet altered fatty acid composition of PL, the effect was not always mirrored in the alterations to composition of sn2. 

## Figures and Tables

**Table 1:** Saturated fatty acid composition of phospholipid (PL), triacylglycerol (TAG), and at sn-2 monoglycerol (sn2) and sn1/3 of fat from the *pars costalis diaphragmatis* (PCD) of beef steers fed dietary oil composed of an equal mixture of flax oil and sunflower oil at 5% of diet and in one of no feed withdrawal (No FW), single feed withdrawal (FW × 1), and feed withdrawal every 8 wk (FW × 4) for 48 h treatments.

Item	Location	Control			Oil			Probability		
		No FW	FW × 1	FW × 4	NoFW	FW × 1	FW × 4	Diet	FW	Diet × FW
Fat (%)^1^		8.10 ± 0.75	8.37 ± 0.82	8.38 ± 0.89	7.59 ± 0.70	6.73 ± 0.78	7.14 ± 0.85	0.090	0.930	0.778

Fatty acid (wt %)^2^										

14:0	PL	1.75 ± 0.15	1.77 ± 0.14	1.60 ± 0.21	1.59 ± 0.20	1.65 ± 0.11	1.52 ± 0.15	0.3646	0.6183	0.9698
	TAG	3.19 ± 0.21	3.34 ± 0.28	2.69 ± 0.16	3.26 ± 0.21	3.32 ± 0.23	3.37 ± 0.28	0.2174	0.4454	0.2914
	sn2	2.35 ± 0.31	2.53 ± 0.29	2.36 ± 0.36	2.78 ± 0.55	2.44 ± 0.59	2.64 ± 0.36	0.5605	0.9813	0.8267
	sn1/3	3.62 ± 0.47	3.74 ± 0.51	2.86 ± 0.27	3.51 ± 0.51	3.65 ± 0.61	3.36 ± 0.67	0.8074	0.4958	0.8013

15:0	PL	1.05 ± 0.09	1.10 ± 0.07	0.93 ± 0.06	0.81 ± 0.07	1.00 ± 0.08	0.90 ± 0.08	0.0545	0.1878	0.3771
	TAG	0.48 ± 0.03	0.58 ± 0.03	0.47 ± 0.04	0.51 ± 0.03	0.55 ± 0.04	0.55 ± 0.03	0.3942	0.0838	0.2742
	sn2	1.51 ± 0.18	1.69 ± 0.29	1.02 ± 0.23	1.54 ± 0.14	1.44 ± 0.27	1.66 ± 0.14	0.4726	0.6098	0.1662
	sn1/3	−0.03 ± 0.08	0.03 ± 0.17	0.19 ± 0.15	−0.01 ± 0.15	0.11 ± 0.14	−0.01 ± 0.08	0.7359	0.6999	0.5304

16:0	PL	26.7 ± 1.51	28.58 ± 1.40	25.61 ± 1.40	21.34 ± 1.78	21.35 ± 1.40	23.16 ± 1.56	0.0001	0.8080	0.2552
	TAG	30.50 ± 0.89	29.09 ± 0.70	28.82 ± 0.81	27.03 ± 0.85	25.33 ± 0.48	26.38 ± 0.81	0.0001	0.1055	0.6427
	sn2	27.45 ± 1.41	28.82 ± 2.28	30.15 ± 2.18	21.34 ± 1.78	28.82 ± 2.27	26.97 ± 2.17	0.5993	0.8669	0.3100
	sn1/3	32.08 ± 1.59	29.23 ± 1.21	28.16 ± 1.72	25.48 ± 1.74	24.65 ± 1.59	26.08 ± 1.35	0.0010	0.4358	0.3523

18:0	PL	18.61 ± 0.82	17.03 ± 1.37	16.50 ± 0.55	19.08 ± 0.69	19.08 ± 0.50	20.39 ± 1.11	0.0041	0.6513	0.1631
	TAG	16.90 ± 1.04	17.65 ± 1.27	15.79 ± 0.86	19.89 ± 1.17	19.83 ± 0.79	20.95 ± 0.85	0.0001	0.9197	0.3220
	sn2	22.78 ± 1.17	23.49 ± 1.36	26.12 ± 1.81	22.72 ± 1.99	22.49 ± 2.23	21.60 ± 2.26	0.2365	0.8098	0.4443
	sn1/3	13.95 ± 1.43	14.81 ± 1.73	10.62 ± 1.94	18.48 ± 2.33	18.50 ± 1.34	20.62 ± 1.58	0.0001	0.8403	0.1599

20:0	PL	0.15 ± 0.02	0.16 ± 0.01	0.18 ± 0.02	0.18 ± 0.02	0.20 ± 0.02	0.20 ± 0.02	0.0307	0.4778	0.7955
	TAG	0.10 ± 0.005	0.12 ± 0.01	0.10 ± 0.01	0.10 ± 0.01	0.11 ± 0.01	1.23 ± 0.01	0.3568	0.2072	0.1070
	sn2	0.96 ± 0.16	1.62 ± 0.31	1.31 ± 0.41	0.70 ± 0.09	1.52 ± 0.32	1.25 ± 0.29	0.5461	0.0391	0.9320
	sn1/3	−0.35 ± 0.08	−0.62 ± 0.16	−0.50 ± 0.20	−0.19 ± 0.04	−0.60 ± 0.15	−0.44 ± 0.14	0.4741	0.0601	0.9036

^1^Reported in He et al. [12] fat content is on as is basis.

^2^sn1/3wt% = (TAGwt% × 3 − sn2MAGwt%)/2 as in Paterson et al. [10], *n* = 12.

**Table 2:** Unsaturated fatty acid composition of phospholipid (PL), triacylglycerol (TAG), and at sn-2 monoglycerol (sn2) and sn1/3 of fat from the *pars costalis diaphragmatis* (PCD) of beef steers fed dietary oil composed of an equal mixture of flax oil and sunflower oil at 5% of diet and in one of no feed withdrawal (No FW), single feed withdrawal (FW × 1), and feed withdrawal every 8 wk (FW × 4) for 48 h treatments.

Item	Location	Control			Oil			Probability		
		No FW	FW × 1	FW × 4	No FW	FW × 1	FW × 4	Diet	FW	Diet × FW
Fat (%)^1^		8.10 ± 0.75	8.37 ± 0.82	8.38 ± 0.89	7.59 ± 0.70	6.73 ± 0.78	7.14 ± 0.85	0.090	0.930	0.778

Fatty acid (wt %)^2^										

16:1c9	PL TAG sn2 sn1/3	3.53 ± 0.27 2.99 ± 0.21 0.89 ± 0.22 4.33 ± 0.59	4.45 ± 0.68 3.14 ± 0.27 0.90 ± 0.25 4.26 ± 0.48	4.67 ± 0.29 3.27 ± 0.26 0.60 ± 0.17 4.61 ± 0.43	3.38 ± 0.30 2.68 ± 0.18 1.22 ± 0.25 3.25 ± 0.46	3.32 ± 0.40 2.75 ± 0.17 1.05 ± 0.16 3.60 ± 0.25	2.92 ± 0.32 2.84 ± 0.19 0.90 ± 0.22 3.61 ± 0.32	0.0042 0.0390 0.1470 0.0133	0.5575 0.6145 0.3476 0.7624	0.1625 0.9587 0.9007 0.8757

18:1t9	PL TAG sn2 sn1/3	0.27 ± 0.05 0.27 ± 0.03 0.41 ± 0.12 0.20 ± 0.08	0.34 ± 0.05 0.22 ± 0.06 0.68 ± 0.19 −0.00 ± 0.12	0.37 ± 0.04 0.22 ± 0.02 0.48 ± 0.15 0.09 ± 0.07	1.24 ± 0.22 0.50 ± 0.06 0.73 ± 0.16 0.52 ± 0.09	1.39 ± 0.27 0.56 ± 0.06 0.81 ± 0.16 0.43 ± 0.08	1.03 ± 0.27 0.48 ± 0.05 0.62 ± 0.14 0.40 ± 0.11	0.0001 0.0001 0.1063 0.0001	0.6047 0.6329 0.3700 0.2974	0.4898 0.5855 0.7840 0.7653

18:1t11	PL TAG sn2 sn1/3	0.60 ± 0.05 0.66 ± 0.10 0.51 ± 0.14 0.73 ± 0.15	0.51 ± 0.07 0.60 ± 0.07 0.72 ± 0.21 0.53 ± 0.16	0.57 ± 0.07 0.56 ± 0.06 0.48 ± 0.15 0.60 ± 0.08	1.00 ± 0.12 1.37 ± 0.19 0.67 ± 0.17 1.72 ± 0.30	1.11 ± 0.07 1.54 ± 0.10 1.13 ± 0.30 1.75 ± 0.20	0.91 ± 0.11 1.16 ± 0.11 0.73 ± 0.20 1.38 ± 0.20	0.0001 0.0001 0.1054 0.0001	0.6818 0.1750 0.1810 0.4750	0.3064 0.3088 0.8312 0.5305

18:1c9	PL TAG sn2 sn1/3	36.27 ± 0.92 40.59 ± 1.07 13.49 ± 0.82 53.87 ± 1.71	35.94 ± 2.26 40.86 ± 1.20 12.97 ± 0.90 54.80 ± 2.06	41.35 ± 0.77 44.10 ± 1.11 13.11 ± 1.14 59.59 ± 1.89	34.16 ± 2.14 40.29 ± 1.47 13.47 ± 0.77 54.35 ± 2.10	35.72 ± 1.13 40.74 ± 0.75 12.36 ± 1.35 53.94 ± 1.42	33.74 ± 0.27 39.48 ± 0.55 14.66 ± 1.30 51.89 ± 1.09	0.0117 0.0606 0.7381 0.0644	0.3050 0.4334 0.5156 0.6027	0.0557 0.0689 0.5819 0.0493

18:2t9c11	PL TAG sn2 sn1/3	0.08 ± 0.02 0.05 ± 0.01 — 0.07 ± 0.01	0.08 ± 0.01 0.06 ± 0.02 — 0.09 ± 0.02	0.09 ± 0.02 0.07 ± 0.01 — 0.10 ± 0.02	0.28 ± 0.03 0.20 ± 0.02 — 0.29 ± 0.03	0.31 ± 0.03 0.26 ± 0.02 — 0.38 ± 0.03	0.23 ± 0.02 0.22 ± 0.03 — 0.33 ± 0.04	0.0001 0.0001 — 0.0001	0.4224 0.1367 — 0.1362	0.1577 0.3201 — 0.3241

18:2*ω*6c9, 12	PL TAG sn2 sn1/3	2.81 ± 0.94 0.92 ± 0.08 3.66 ± 0.56 −0.45 ± 0.26	1.31 ± 0.38 0.95 ± 0.07 2.92 ± 0.34 −0.04 ± 0.20	0.98 ± 0.18 0.80 ± 0.06 3.27 ± 0.38 −0.43 ± 0.22	5.05 ± 1.94 1.02 ± 0.10 3.16 ± 0.41 −0.05 ± 0.25	4.63 ± 1.49 1.20 ± 0.12 3.03 ± 0.27 0.29 ± 0.28	3.81 ± 1.68 1.04 ± 0.17 2.81 ± 0.43 0.16 ± 0.31	0.0103 0.0262 0.4018 0.0406	0.4857 0.3416 0.5291 0.3348	0.9137 0.7200 0.7168 0.8670

18:3*ω*3C9, 12, 15	PL TAG sn2 sn1/3	0.26 ± 0.05 0.27 ± 0.02 1.55 ± 0.25 −0.37 ± 0.11	0.21 ± 0.09 0.26 ± 0.02 1.09 ± 0.12 −0.16 ± 0.07	0.22 ± 0.01 0.25 ± 0.02 0.99 ± 0.20 −0.08 ± 0.10	0.30 ± 0.07 0.36 ± 0.04 0.99 ± 0.17 0.04 ± 0.12	0.43 ± 0.09 0.48 ± 0.04 1.22 ± 0.12 0.11 ± 0.09	0.30 ± 0.09 0.35 ± 0.05 0.99 ± 0.20 0.03 ± 0.14	0.0326 0.0001 0.4591 0.0050	0.6321 0.1043 0.2189 0.3475	0.3615 0.1182 0.1301 0.3903

CLAc9t11	PL TAG sn2 sn1/3	0.19 ± 0.05 0.34 ± 0.04 1.70 ± 0.50 −0.35 ± 0.26	0.21 ± 0.06 0.27 ± 0.03 1.49 ± 0.39 −0.34 ± 0.19	0.16 ± 0.03 0.32 ± 0.03 1.61 ± 0.42 −0.32 ± 0.21	0.31 ± 0.05 0.46 ± 0.04 1.13 ± 0.25 0.13 ± 0.15	0.36 ± 0.06 0.69 ± 0.06 1.17 ± 0.31 0.45 ± 0.15	0.31 ± 0.07 0.41 ± 0.08 2.07 ± 0.54 −0.33 ± 0.25	0.0034 0.0001 0.6811 0.0177	0.5983 0.0665 0.4191 0.1903	0.9584 0.0022 0.4420 0.1687

CLAt10c12	PL TAG sn2 sn1/3	0.02 ± 0.01 0.02 ± 0.00 1.60 ± 0.48 −0.77 ± 0.24	0.04 ± 0.01 0.02 ± 0.00 2.06 ± 0.52 −1.00 ± 0.26	0.02 ± 0.01 0.02 ± 0.00 2.13 ± 0.53 −1.04 ± 0.26	0.02 ± 0.01 0.02 ± 0.00 1.35 ± 0.47 −0.65 ± 0.24	0.02 ± 0.01 0.03 ± 0.00 1.56 ± 0.46 −0.74 ± 0.23	0.02 ± 0.01 0.02 ± 0.00 2.59 ± 0.51 −1.25 ± 0.26	0.2976 0.3563 0.8140 0.7797	0.3666 0.1712 0.2088 0.2140	0.5446 0.3232 0.6164 0.6239

^1^Reported in He et al. [12] fat content is on as is basis.

^2^sn1/3wt% = (TAGwt% × 3 − sn2MAGwt%)/2 as in Paterson et al. [10], *n* = 12.

**Table 3:** Saturated fatty acid composition of phospholipid (PL), triacylglycerol (TAG), and at sn-2 monoglycerol (sn2) and sn1/3 of fat from the subcutaneous (SQ) fat of beef steers fed dietary oil composed of an equal mixture of flax oil and sunflower oil at 5% of diet and in one of no feed withdrawal (No FW), single feed withdrawal (FW × 1), and feed withdrawal every 8 wk (FW × 4) for 48 h treatments.

Item	Location	Control			Oil			Probability		
		No FW	FW × 1	FW × 4	No FW	FW × 1	FW × 4	Diet	FW	Diet × FW
Fat (%)^1^		82.63 ± 1.93	79.19 ± 2.49	80.85 ± 1.74	88.11 ± 2.27	81.89 ± 1.95	82.28 ± 1.93	0.061	0.054	0.597

Fatty acid (wt %)^2^										

14:0	PL TAG sn2 sn1/3	5.42 ± 0.64 3.19 ± 0.24 4.38 ± 1.33 4.87 ± 1.06	4.86 ± 0.63 3.34 ± 0.28 2.74 ± 1.12 4.34 ± 1.13	4.71 ± 0.44 2.69 ± 0.16 3.69 ± 1.16 3.21 ± 0.68	5.72 ± 0.68 3.26 ± 0.21 3.89 ± 1.35 4.68 ± 0.81	6.68 ± 0.90 3.32 ± 0.23 2.03 ± 0.21 5.15 ± 0.46	5.41 ± 0.83 3.37 ± 0.28 3.48 ± 1.07 4.47 ± 0.68	0.1082 0.2174 0.6151 0.3483	0.5857 0.4454 0.3002 0.4109	0.5357 0.2914 0.9759 0.6573

15:0	PL TAG sn2 sn1/3	0.80 ± 0.08 0.48 ± 0.03 1.39 ± 0.19 0.24 ± 0.12	0.77 ± 0.05 0.58 ± 0.03 1.24 ± 0.14 0.23 ± 0.07	0.77 ± 0.05 0.47 ± 0.04 1.09 ± 0.13 0.32 ± 0.10	0.79 ± 0.05 0.51 ± 0.03 1.39 ± 0.16 0.21 ± 0.08	0.95 ± 0.10 0.55 ± 0.04 1.29 ± 0.21 0.30 ± 0.13	0.83 ± 0.11 0.55 ± 0.03 1.17 ± 0.11 0.24 ± 0.12	0.2361 0.3942 0.7374 0.8346	0.6591 0.0838 0.2832 0.5576	0.4672 0.2742 0.9712 0.8758

16:0	PL TAG sn2 sn1/3	29.14 ± 1.69 30.50 ± 0.89 25.06 ± 1.63 29.99 ± 1.58	32.67 ± 2.44 29.09 ± 0.70 22.89 ± 1.46 32.72 ± 1.42	27.83 ± 0.47 28.82 ± 0.81 28.38 ± 2.92 26.38 ± 1.53	26.68 ± 1.62 27.03 ± 0.85 25.73 ± 1.59 28.55 ± 1.61	24.45 ± 1.28 25.33 ± 0.48 23.02 ± 1.11 28.01 ± 1.27	25.17 ± 1.67 26.38 ± 0.64 21.61 ± 1.90 27.44 ± 1.23	0.0022 0.0001 0.1950 0.1583	0.4632 0.1055 0.3781 0.0601	0.1627 0.6427 0.0929 0.1461

18:0	PL TAG sn2 sn1/3	9.04 ± 0.42 16.90 ± 1.04 19.19 ± 1.97 8.53 ± 1.56	11.11 ± 0.93 17.65 ± 1.27 21.74 ± 2.14 9.05 ± 1.66	9.28 ± 0.51 15.79 ± 0.86 25.15 ± 2.70 4.06 ± 1.85	12.30 ± 0.89 19.89 ± 1.17 23.81 ± 1.52 10.47 ± 1.81	12.02 ± 0.48 19.83 ± 0.79 21.84 ± 2.65 11.63 ± 1.91	13.44 ± 0.83 20.95 ± 0.85 17.63 ± 2.01 13.83 ± 1.81	0.0001 0.0001 0.6076 0.0019	0.4269 0.9197 0.9826 0.7287	0.0651 0.3220 0.0275 0.0521

20:0	PL TAG sn2 sn1/3	0.15 ± 0.03 0.10 ± 0.01 1.03 ± 0.24 −0.40 ± 0.12	0.13 ± 0.01 0.12 ± 0.01 1.56 ± 0.40 0.50 ± 0.13	0.11 ± 0.02 0.10 ± 0.01 1.41 ± 0.29 0.59 ± 0.15	0.12 ± 0.02 0.10 ± 0.01 1.67 ± 0.45 −0.49 ± 0.11	0.13 ± 0.02 0.11 ± 0.01 1.54 ± 0.26 −0.65 ± 0.14	0.14 ± 0.02 0.12 ± 0.01 1.17 ± 0.22 0.45 ± 0.11	0.8781 0.3568 0.6247 0.7545	0.9652 0.2072 0.7249 0.6306	0.3151 0.1070 0.3594 0.2144

^1^Reported in He et al. [12] fat content is on as is basis.

^2^sn1/3wt% = (TAGwt% × 3 − sn2MAGwt%)/2 as in Paterson et al. [10], *n* = 12.

**Table 4:** Unsaturated fatty acid composition of phospholipid (PL), triacylglycerol (TAG), and at sn-2 monoglycerol (sn2) and sn1/3 of fat from the subcutaneous (SQ) fat of beef steers fed dietary oil composed of an equal mixture of flax oil and sunflower oil at 5% of diet and in one of no feed withdrawal (No FW), single feed withdrawal (FW × 1) and feed withdrawal every 8 wk (FW × 1), for 48 h treatments.

Item	Location	Control			Oil			Probability		
		No FW	FW × 1	FW × 4	No FW	FW × 1	FW × 4	Diet	FW	Diet × FW
Fat (%)^1^		82.63 ± 1.93	79.19 ± 2.49	80.85 ± 1.74	88.11 ± 2.27	81.89 ± 1.95	82.28 ± 1.93	0.061	0.054	0.597

Fatty acid (wt%)^2^										

16:1c9	PL TAG sn2 sn1/3	9.58 ± 1.20 2.99 ± 0.21 1.40 ± 0.28 6.49 ± 0.42	7.64 ± 1.14 3.14 ± 0.27 1.10 ± 0.12 5.44 ± 0.60	8.65 ± 0.82 3.27 ± 0.26 0.98 ± 0.15 6.61 ± 0.37	6.65 ± 1.08 2.68 ± 0.18 1.15 ± 0.11 5.26 ± 0.48	7.76 ± 0.50 2.75 ± 0.17 1.27 ± 0.18 5.54 ± 0.54	7.26 ± 0.74 2.84 ± 0.18 1.11 ± 0.25 5.49 ± 0.37	0.0712 0.0390 0.9313 0.0595	0.9055 0.6145 0.4987 0.4853	0.2788 0.9587 0.4767 0.3008

18:1t9	PL TAG sn2 sn1/3	0.22 ± 0.04 0.27 ± 0.03 0.68 ± 0.11 −0.13 ± 0.12	0.21 ± 0.03 0.22 ± 0.06 0.94 ± 0.18 −0.14 ± 0.10	0.24 ± 0.05 0.22 ± 0.02 0.43 ± 0.09 0.14 ± 0.05	0.43 ± 0.06 0.50 ± 0.06 0.67 ± 0.14 0.16 ± 0.11	0.48 ± 0.05 0.56 ± 0.06 0.56 ± 0.08 0.44 ± 0.08	0.41 ± 0.01 0.48 ± 0.05 0.72 ± 0.15 0.33 ± 0.10	0.0001 0.0001 0.4767 0.0001	0.8844 0.6329 0.4033 0.0912	0.5295 0.5855 0.0420 0.1378

18:1t11	PL TAG sn2 sn1/3	0.56 ± 0.08 0.66 ± 0.10 1.21 ± 0.12 0.52 ± 0.16	0.50 ± 0.05 0.60 ± 0.07 0.80 ± 0.12 0.45 ± 0.11	0.54 ± 0.07 0.56 ± 0.06 0.48 ± 0.09 0.77 ± 0.22	1.06 ± 0.07 1.37 ± 0.19 0.84 ± 0.13 1.28 ± 0.21	1.02 ± 0.05 1.54 ± 0.10 0.66 ± 0.11 1.61 ± 0.24	0.90 ± 0.06 1.16 ± 0.11 1.14 ± 0.15 1.17 ± 0.15	0.0001 0.0001 0.6333 0.0001	0.3995 0.1750 0.0525 0.7857	0.3902 0.3088 0.0003 0.1421

18:1c9	PL TAG sn2 sn1/3	32.34 ± 1.22 40.59 ± 1.07 15.5 ± 1.76 55.38 ± 2.59	33.80 ± 1.71 40.86 ± 1.20 14.13 ± 1.37 58.26 ± 1.86	38.06 ± 0.88 44.10 ± 1.11 11.63 ± 1.32 61.94 ± 1.82	33.56 ± 0.92 40.29 ± 1.47 14.63 ± 1.01 53.14 ± 2.25	33.27 ± 1.56 40.74 ± 0.75 17.11 ± 2.40 53.99 ± 2.11	36.13 ± 1.82 39.48 ± 0.55 13.08 ± 1.37 55.86 ± 1.83	0.7197 0.0606 0.3845 0.0178	0.0095 0.4334 0.1160 0.0937	0.5346 0.0689 0.4930 0.6576

18:2t9c11	PL TAG sn2 sn1/3	0.14 ± 0.03 0.05 ± 0.01 — 0.23 ± 0.05	0.10 ± 0.02 0.06 ± 0.01 — 0.15 ± 0.02	0.14 ± 0.02 0.07 ± 0.01 — 0.16 ± 0.02	0.39 ± 0.07 0.20 ± 0.02 — 0.50 ± 0.06	0.55 ± 0.10 0.26 ± 0.02 — 0.51 ± 0.06	0.41 ± 0.07 0.22 ± 0.03 — 0.52 ± 0.05	0.0001 0.0001 — 0.0001	0.5147 0.1367 — 0.7085	0.1567 0.3201 — 0.4597

18:2*ω*6c9,12	PL TAG sn2 sn1/3	1.55 ± 0.30 0.92 ± 0.08 2.66 ± 0.34 0.39 ± 0.24	1.38 ± 0.18 0.95 ± 0.07 2.32 ± 0.18 0.38 ± 0.15	1.54 ± 0.20 0.80 ± 0.06 1.66 ± 0.18 0.80 ± 0.16	1.52 ± 0.22 1.02 ± 0.10 2.04 ± 0.17 0.65 ± 0.13	1.98 ± 0.27 1.20 ± 0.12 2.5 ± 0.14 0.58 ± 0.11	1.59 ± 0.29 1.04 ± 0.17 2.5 ± 0.29 0.49 ± 0.23	0.3099 0.0262 0.4768 0.7545	0.8278 0.3416 0.3905 0.6306	0.3844 0.7200 0.0079 0.2144

18:3*ω*3C9,12,15	PL TAG sn2 sn1/3	0.48 ± 0.06 0.27 ± 0.02 1.13 ± 0.16 0.10 ± 0.10	0.42 ± 0.06 0.26 ± 0.02 1.22 ± 0.18 −0.16 ± 0.10	0.56 ± 0.08 0.25 ± 0.02 0.83 ± 0.17 0.18 ± 0.10	0.74 ± 0.12 0.36 ± 0.04 0.49 ± 0.09 0.41 ± 0.10	0.95 ± 0.13 0.48 ± 0.04 0.76 ± 0.09 0.43 ± 0.09	0.68 ± 0.10 0.35 ± 0.05 1.17 ± 0.10 0.18 ± 0.09	0.0002 0.0001 0.0330 0.0005	0.6768 0.1043 0.3114 0.4599	0.0984 0.1182 0.0021 0.0153

CLAc9t11	PL TAG sn2 sn1/3	0.83 ± 0.12 0.34 ± 0.04 2.07 ± 0.61 −0.29 ± 0.29	0.68 ± 0.12 0.27 ± 0.03 2.43 ± 0.48 −0.53 ± 0.21	1.03 ± 0.15 0.32 ± 0.03 1.47 ± 0.37 0.36 ± 0.24	1.25 ± 0.21 0.46 ± 0.04 1.44 ± 0.32 0.82 ± 0.17	1.81 ± 0.20 0.69 ± 0.06 2.07 ± 0.33 0.66 ± 0.16	1.03 ± 0.20 0.41 ± 0.08 3.00 ± 0.71 0.02 ± 0.40	0.0001 0.0001 0.8656 0.0050	0.4377 0.0665 0.9664 0.7646	0.0475 0.0022 0.0163 0.0088

CLAt10c12	PL TAG sn2 sn1/3	0.003 ± 0.00 0.02 ± 0.00 2.65 ± 0.59 −1.29 ± 0.30	0.001 ± 0.00 0.02 ± 0.00 2.22 ± 0.60 −1.07 ± 0.30	0.02 ± 0.01 0.02 ± 0.00 1.42 ± 0.46 −0.65 ± 0.23	0.03 ± 0.02 0.02 ± 0.00 1.21 ± 0.38 0.54 ± 0.19	0.10 ± 0.03 0.03 ± 0.00 1.50 ± 0.34 −0.68 ± 0.17	0.06 ± 0.02 0.03 ± 0.00 3.05 ± 0.68 −0.58 ± 0.12	0.0002 0.3563 0.6931 0.6213	0.1797 0.1712 0.7628 0.6213	0.1041 0.3232 0.0174 0.0155

^1^Reported in He et al. [12] fat content is on as is basis.

^2^sn1/3wt% = (TAGwt% × 3 − sn2MAGwt%)/2 as in Paterson et al. [10], *n* = 12.

**Table 5:** Eicosapentaenoic acid (EPA) and Docosahexaenoic acid (DHA) composition of phospholipid (PL), triacylglycerol (TAG), and at sn-2 monoglycerol (sn2) and sn1/3 of fat from the *pars costalis diaphragmatis* (PCD) and subcutaneous fat (SQ) of beef steers fed dietary oil composed of an equal mixture of flax oil and sunflower oil at 5% of diet and in one of no feed withdrawal (No FW), single feed withdrawal (FW × 1), and feed withdrawal every 8 wk (FW × 4) for 48 h treatments.

Item	Location	Control			Oil			Probability		
		No FW	FW × 1	FW × 4	No FW	FW × 1	FW × 4	Diet	FW	Diet × FW
PCD										

EPA (wt%)	PL TAG sn2 sn1/3	0.12 ± 0.05 0.004 ± 0.00 0.98 ± 0.30 −0.48 ± 0.15	0.04 ± 0.01 0.003 ± 0.00 1.11 ± 0.28 −0.54 ± 0.14	0.05 ± 0.01 0.00 ± 0.00 1.11 ± 0.31 −0.56 ± 0.16	0.12 ± 0.05 0.01 ± 0.00 0.99 ± 0.26 −0.48 ± 0.13	0.14 ± 0.05 0.00 ± 0.00 1.18 ± 0.34 −0.59 ± 0.17	0.13 ± 0.07 0.01 ± 0.00 0.88 ± 0.26 −0.42 ± 0.13	0.1187 0.0770 0.8375 0.8317	0.7509 0.5379 0.8203 0.8283	0.4996 0.4211 0.8609 0.8195
DHA (wt%)	PL TAG sn2 sn1/3	0.17 ± 0.10 0.004 ± 0.00 1.39 ± 0.26 −0.67 ± 0.14	0.07 ± 0.01 0.01 ± 0.01 0.83 ± 0.25 0.40 ± 0.12	0.24 ± 0.12 0.00 ± 0.00 1.03 ± 0.28 −0.36 ± 0.09	0.17 ± 0.08 0.01 ± 0.00 0.99 ± 0.26 −0.48 ± 0.13	0.21 ± 0.09 0.01 ± 0.00 1.25 ± 0.19 −0.59 ± 0.10	0.16 ± 0.08 0.02 ± 0.01 1.60 ± 0.47 −0.58 ± 0.17	0.8727 0.2593 0.4054 0.4733	0.8110 0.3560 0.6429 0.6922	0.4051 0.0700 0.1939 0.2150

SQ										

EPA (wt%)	PL TAG sn2 sn1/3	0.09 ± 0.04 0.01 ± 0.00 1.76 ± 0.52 −0.87 ± 0.26	0.04 ± 0.03 0.002 ± 0.00 1.33 ± 0.40 −0.66 ± 0.20	0.05 ± 0.05 0.00 ± 0.00 1.18 ± 0.28 −0.56 ± 0.16	0.06 ± 0.02 0.03 ± 0.00 1.35 ± 0.31 −0.67 ± 0.15	0.10 ± 0.03 0.003 ± 0.00 1.11 ± 0.26 −0.55 ± 0.13	0.07 ± 0.02 0.01 ± 0.00 2.76 ± 0.68 −1.25 ± 0.36	0.3893 0.5538 0.6681 0.4934	0.8285 0.8194 0.5088 0.4209	0.2728 0.4455 0.1804 0.1026
DHA (wt%)	PL TAG sn2 sn1/3	0.10 ± 0.03 0.02 ± 0.02 1.21 ± 0.30 −0.57 ± 0.16	0.08 ± 0.03 0.03 ± 0.01 1.64 ± 0.40 −0.78 ± 0.21	0.11 ± 0.02 0.02 ± 0.00 0.74 ± 0.18 −0.35 ± 0.08	0.10 ± 0.03 0.03 ± 0.00 0.65 ± 0.14 −0.29 ± 0.08	0.10 ± 0.03 0.01 ± 0.00 1.44 ± 0.27 −0.71 ± 0.13	0.11 ± 0.03 0.03 ± 0.01 2.09 ± 0.61 −0.79 ± 0.22	0.7232 0.9240 0.5004 0.8162	0.8102 0.9113 0.2041 0.1331	0.9032 0.4261 0.0225 0.0714

^1^sn1/3wt% = (TAGwt% × 3 − sn2MAGwt%)/2 as in Paterson et al. [10], *n  *= 12.

**Table 6:** Summary table of the principal location of occurrence of the fatty acids in fat from the *pars costalis diaphragmatis* (PCD) muscle and the subcutaneous (SQ) fat from the brisket of steers fed diets without or with n6 and n3 fatty acids.

Tissue	Phospholipid	Triacylglycerol	Sn2	Sn1/3
PCD	15:0		15:0	
			18:0	
			20:0	
				16:1c9
			18:1t9	
		18:1c9		18:1c9
	18:2*ω*6		18:2*ω*6	
			18:3*ω*3	
		CLAc9t11	CLAc9t11	
			CLAc10t12	
	EPA		EPA	
	DHA		DHA	

SQ	15:0		15:0	
		18:0	18:0	
			20:0	
	16:1c9			16:1c9
			18:1t9	
		18:1c9		18:1c9
	18:2*ω*6		18:2*ω*6	
	18:3*ω*3		18:3*ω*3	
	CLAc9t11		CLAc9t11	
	CLAc10t12		CLAc10t12	
	EPA		EPA	
	DHA		DHA	

Fatty acids represented by number of carbons: number of double bonds in either the cis (c) or trans (t) position.
